# Incidence, pattern and mechanisms of injuries and fractures in children under two years of age: a population-based study

**DOI:** 10.1186/s12891-026-09886-8

**Published:** 2026-05-04

**Authors:** Rien Fredrik Ragnar Avenarius, Edvard Johan Enoksen, Karen Rosendahl

**Affiliations:** 1 Department of Clinical Medicine, UiT the Artic University of Norway, Tromsø, Norway; 2 Department of Radiology, University Hospital of North Norway, Pb 100, Tromsø, 9038 Norway

**Keywords:** Fracture, Infant, Child, Incidence

## Abstract

**Background:**

Epidemiological data on injuries and fractures in children under two years of age is limited, thus, we aimed to explore the incidence, patterns and mechanisms.

**Methods:**

Retrospective, population-based cross-sectional study including children under two, seen at the A&E department due to a trauma, or having a radiograph due to birth trauma. All radiographs were re-reviewed.

**Results:**

Four hundred thirty children (49.8% female), mean age 14.7 months (SD 6.8), were included, of whom four had a high-energy trauma and 42 were birth-related. Of 388 children (50.3% female) (mean age 16.3 months, SD 5.1 months) with non-birth related injury, 163 (42.0%) had a fracture (annual incidence 4.3 per 1,000 children; 2.4 per 1,000 infants vs. 6.1 per 1,000 children aged 12 to 24 months of age. 47/163 (28.8%) fractures involved the forearm and 41 (25.2%) involved the leg. Fracture mechanisms 39.9% fall from more than own height/furniture; 17.2% fall from own height, 6.7% crush injury and 6.1% dropped by parent. In 12.9% of the traumas, no mechanism was provided.

The number of fractures increased significantly by age group (*p*=0.027), and the distribution differed, with skull fractures predominating in 0-6-months-old (33.0%), clavicle fractures (33.0%) in 6-12-months-old and forearm fractures in the two older age groups (35.0% and 34.4%, respectively) (*p* < 0.001). No classic metaphyseal lesions were found in the entire cohort. Four children, all of whom had radiological “red flags”, had inflicted injury.

Forty-two children had birth-related injury, of whom 50.0% had a fracture (1.1 per 1,000 live births).

**Conclusions:**

Fractures in infants are rare, in particular classic metaphyseal fractures. The occurrence of radiological “red flags” should raise suspicion of non-accidental injury and instigate further assessment.

## Background

Injuries and fractures in children are relatively frequent, with a yearly fracture incidence of 18–25 per 1,000 children under the age of 16 years [1, 2]. Except for children under the age of two, the incidence is higher in boys than in girls, increasing with age until puberty [1]. However, data on the incidence and types of injuries and fractures in children under two years of age are limited [3]. A recent systematic review identified only 12 studies of moderate to good quality, showing an overall annual incidence rate of 5.3 to 9.5 fractures per 1,000 children aged 0–2 years [4]. Based on 407 patients from three of the 12 studies, the most common sites were the radius and ulna (25 to 40% of the cases), followed by the tibia and fibula (up to 28%) and the clavicle (approximately 15%) [4].

In infants, data from three of the 12 studies indicate an incidence, ranging from 0.7 to 4.6 per 1,000, with fractures most frequently affecting the clavicle and the proximal humerus in around one fifth of cases [4]. Notably, only one metaphyseal lesion, a lesion which is often associated with inflicted injury [5], was reported [4, 6]. The fracture mechanism, as reported in two of the studies, was a fall from low height in 50–70%, whilst in five studies the authors concluded that more studies were warranted to determine the more common injury patterns, so that differences from abusive trauma may be better understood [4].

The aim of this study was to examine the incidence, type and mechanism of injuries and fractures, including birth injury and high energy trauma, in a population-based cohort of children under two years of age. In addition, we estimated the occurrence of suspected inflicted injury, and its association with radiological “red flags”.

## Methods

This retrospective, population-based cross-sectional study was carried out using patient data from the University Hospital North Norway (UNN), being the only Accident and Emergency (A&E) department serving the population under investigation. The study included all children under the age of two years who presented at the A&E department due to trauma warranting radiographic examination between January 1st, 2010, and December 31st, 2023, or newborns having a radiograph due to a birth trauma. Exclusion criteria were children with a known bone disease associated with increased fracture risk, such as osteogenesis imperfecta or metabolic bone disease, those with registered postal codes outside the investigated municipalities. Patients were identified through searches in the PACS system (Picture, Archiving and Communication System) (Sectra IDS7) at the radiology department. Additional clinical data were retrieved from the hospital information system (DIPS arena). The following data were recorded: demographics variables (age, gender, postal code (grouped into rural or urban areas), the number of days between the trauma and the first radiographic examination, mechanism of injury as provided by the caretaker(s), whether or not it was a birth injury, whether an additional skeletal survey was performed, and in the event of a skeletal survey, if there were any findings. We also registered the total number of visits to the A&E, including the total number of new fractures. The first 300 radiological examinations were reviewed in consensus by three investigators (RA, EJE, KR) of whom the latter had more than 35 years of experience in pediatric musculoskeletal radiology, whilst the two others were medical students. Potential classic metaphyseal lesions (CMLs) were given particular attention during the reading sessions. The remaining 130 examinations were reviewed by two of the investigators (RA, EJE) with the abovementioned senior consultant (KR) available in equivocal cases. The following features were registered: fracture (yes/no), fracture location and type (complete, incomplete (bowing/greenstick/buckle-torus), fissure, avulsion, classic metaphyseal injury (CML), inconclusive/not defined), fracture mechanism as provided by the caretaker and whether there were any signs of fracture healing, such as subperiosteal new bone formation (SPNBF), callus or sclerosis. SPBNF was defined as new bone paralleling the original cortex of the bone with a linear configuration and callus was defined as mineralization first evident as amorphous opacity near the cortical margins of the fracture, and subsequently progressing centrifugally away from the injury site with a more spherical configuration, as suggested by Walters et al. [7].

A fissure was defined as a lytic line within the bone with no visible involvement of the cortex, confirmed by healing signs at follow-up. Children with a negative radiograph, but with clinical suspicion of a fracture, had a follow-up radiograph performed after approximately 10 days. If any healing signs at follow-up, this was classified as a fissure. Paired fractures to the radius and ulna or tibia and fibula were registered as forearm/antebrachium or leg fracture, respectively. Injury mechanism was grouped into a) falling (from own height, over own height and less than 3 m (typically fall from furniture), dropped by parent or fall from unknown height), b) crush injury, c) trampoline injury, d) stretch/pull, e) high energy trauma, f) unknown / unwitnessed or g) no information on mechanism provided in the clinical journal. High energy traumas were defined as falls from more than three meters height, road traffic accidents, or accidents involving major trauma to the patient. For the purpose of the current study, red flags for non-accidental injury were defined as a finding of fracture healing signs on the radiograph taken at presentation, inability of the caretaker to present a reasonable injury mechanism, and an interval of two days or more before seeking medical advice. The two-days interval was used as a surrogate for specific information on significance and reason for the delay. A triple Venn diagram was used to identify sheared features known to be associated with inflicted injury.

The project was approved by REK-North (Regional Committee for Medical and Health Research Ethics, 966951), and a confidentiality waiver under Sect.  29 of the Health Personnel Act was obtained as the study was deemed to be of significant public interest. Data handling and storage were performed in accordance with the relevant guidelines and regulations and approved by the local data protection officer (PVO; 2024/4625-2). The radiographic images presented are anonymized examples from prior clinical cases, independent of the study cohort, and do not contain identifiable information.

Descriptive statistics were used to summarize data. Incidence rates were calculated based on the number of children / fractures, by the annual number of children under two years of age residing in the area/municipalities under investigation (Statistics Norway (https://www.ssb.no/befolkning). Differences in the number of fractures between males and females, between urban and rural residents, between seasons, and fracture type, localisation and injury mechanism by age group (0–6 months, 6–12 months, 12–18 months, 18–24 months) were examined using Chi-Square test (2-sided) or Fisher’s exact test as appropriate. A *p*-value < 0.05 was considered statistically significant. Statistical analyses were performed using Predictive Analytics Software (SPSS) version 28 (IBM, Armonk, NY).

## Results

**Fig. 1:**
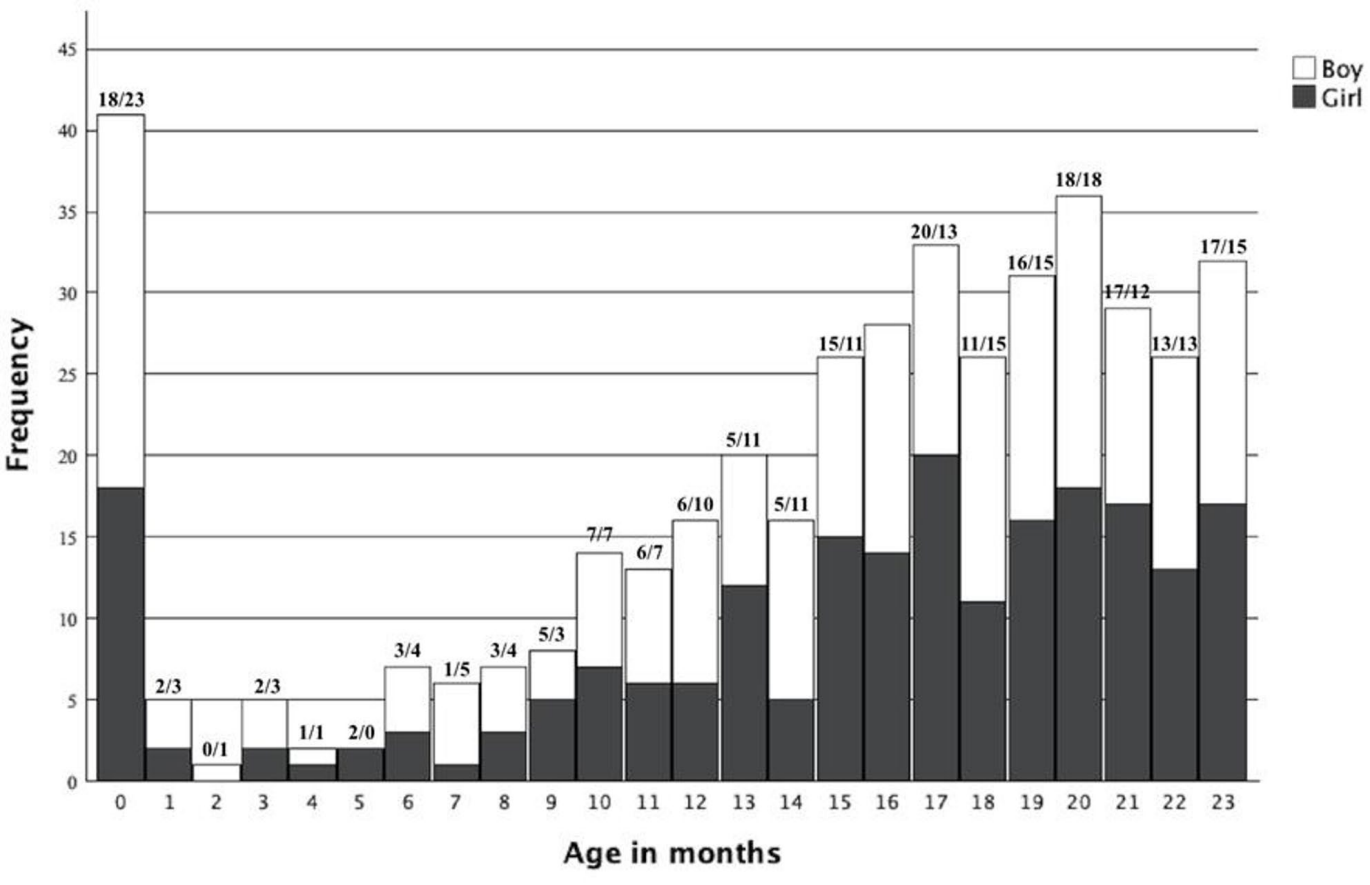
Four hundred thirty children under the age of two years, attending the A&E department for an injury between 2010 and 2024, by age (months) and gender. The numbers above the columns represent boys/girls

A total of 430 children (49.8% female) with a mean age of 14.7 months (SD 6.8) were included (Fig. 1). Among them, four children (25% female) sustained a high-energy trauma and 42 (45.2% female) sustained a birth related trauma. No significant differences in the number of injuries (*p* = 0.76) or fractures (*p* = 0.84) were observed according to gender, therefore, further results were merged.

### Non-birth-related injuries / fractures

**Fig. 2:**
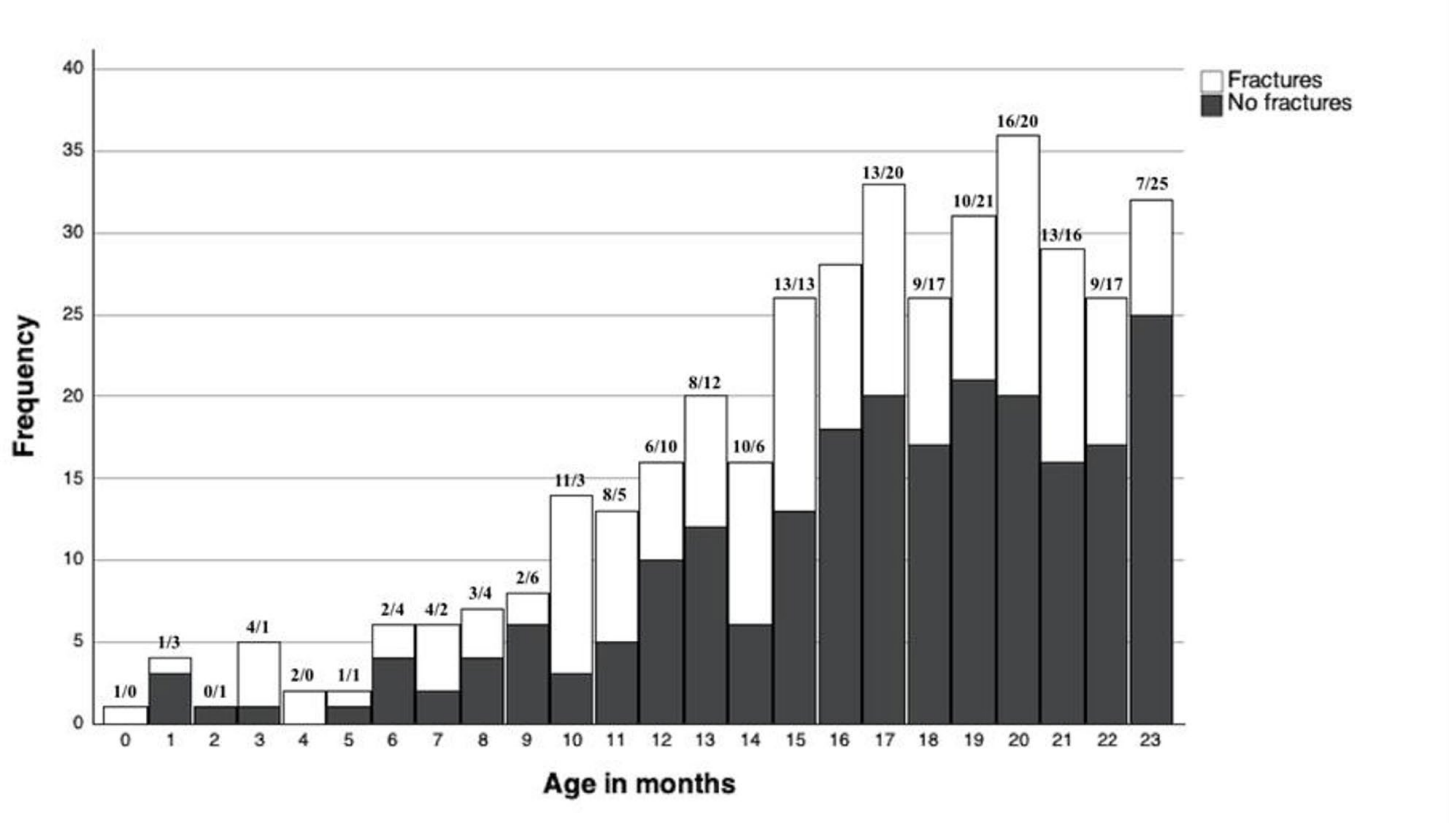
Number of injuries by age (in months) in a cohort of 388 children under 2 years of age (birth injuries excluded). The numbers above each column represent fracture / no fracture

Of 388 children (50.3% female) (mean age 16.3 months, SD = 5.1 months) with a non-birth-related injury, 163 (42.0%) had a fracture (Fig. 2), yielding an annual fracture incidence of 4.3 per 1,000 children under the age of two. Infants had a significantly lower annual incidence as compared to children between 12 and 24 months of age (2.4 per 1,000 vs. 6.1 per 1,000, *p* = 0.01).

Except for those sustaining an antebrachium fracture, or a fracture to both leg-bones, four children (1.0%) sustained two fractures during the same trauma, while two out of 388 children (0.5%) experienced more than one trauma resulting in a fracture before the age of two years. Children living in urban areas (*n* = 320) had a higher annual fracture incidence as compared to those living in rural areas (*n* = 68); 5.2 vs. 2.5 per 1,000 (*p* < 0.001). Finally, no significant differences in the occurrence of fractures were observed according to seasonality, with 38 fractures sustained in winter, 36 in spring, 53 in summer, and 36 in fall, *p* = 0.28.

### Injury and fracture mechanisms

Injury and fracture mechanisms by age group for 388 non-birth related injuries are given in Table 1. The three most common injury mechanisms were fall from more than own height / furniture, followed by falls from own height and crush injuries (Table 1). In 52 (13.4%) of the injuries, no mechanism was provided by the caretaker (unwitnessed). In 8 (2.1%) of the cases, no information on mechanism was available in the medical notes.

**Table 1:** Injury and fracture mechanism in 388 0-2-year-olds attending the A&E due to trauma, birth injuries excluded, by age group during 2010–2024. Proportion of injuries causing a fracture is given in parenthesis

Injury mechanism	0–6 months	6–12 months	12–18 months	18–24 months	Total number of injuries (% with fractures)
Fall from more than own height / furniture	5	20	26	42	93 (69.9)
Fall from own height	0	8	26	33	67 (41.8)
Contusion	0	1	8	5	14 (7.1)
Stretch/pull	0	4	14	23	41 (9.8)
Trampoline	0	0	3	13	16 (43.8)
Dropped by parent	4	5	6	1	16 (62.5)
Torsion	0	0	2	1	3 (66.7)
Crush injury	0	8	27	26	61 (18.0)
Fall from unknown height	0	0	4	8	12(50.0)
High energy trauma	0	2	2	0	4 (75.0)
Unknown / unwitnessed	4	4	19	25	52 (40.4)
Other (burn injury) and	0	0	0	1	1 (0)
No information available	2	2	2	2	8 (62.5)
Total (%)	15 (3.9)	54 (13.9)	139 (35.8)	180 (46.4)	388 (42)

Of the different injury mechanisms, the following caused the higher fracture rates, in declining order; fall from more than own height/furniture 65/93 (69.9%), dropped by parent 10/16 (62.5%), fall on/from trampoline 7/16 (43.8%), fall from own height 28/67 (41.8%) and unknown (unwitnessed) mechanisms 21/52 (40.4%) (Table 1).

Seven trampoline injuries resulted in a fracture, all involving the tibia, of which three were proximal, two were distal and two involved the shaft.

The injury mechanism differed according to age, with falls from more than own height/furniture predominating in infants and fall from own height and crush injuries predominating in children 12–24 months of age (*p* < 0.001) (Table 1).

The reported mechanism for the 163 fractures by location is listed in Table 2. Of the 46 fractures involving the forearm, 20 (43.5%) were caused by a fall from more than own height/furniture, while 13 (28.3%) were caused by a fall from own height. 41 fractures involved the leg, of which 16 (39.0%) were caused by a fall from more than own height/furniture and seven by trampoline. Of a total of 10 femur fractures (incidence rate 0.3 per 1,000), three were sustained because the child was dropped by a parent, four were due to fall from more than own height/furniture, one was due to stretching/pulling, one to torsion and one was unknown (Table 2).

**Table 2:**
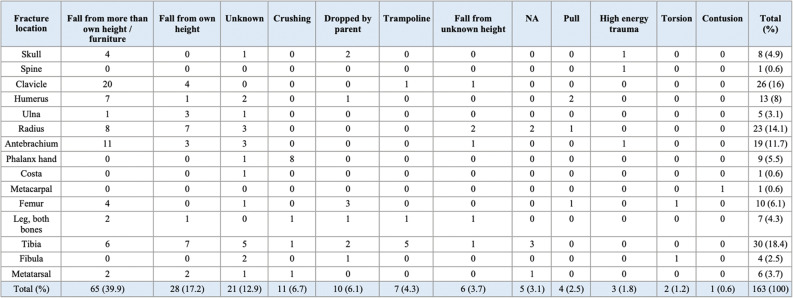
Reported fracture mechanisms of 163 fractures by localization, in a population-based cohort of 388 children 0-2 years of age. Birth related injuries excluded

### Fractures by location and age group

The number of fractures by localization and age group is listed in Table 3. Of a total of 163 fractures, 47 (28.8%) involved the forearm, of which 23 were isolated fractures to the radius, five were fractures to the ulna alone and 19 were two-bone, or antebrachium fractures. There were 41 (25.2%) fractures to the leg, of which 30 involved the tibia, four involved the fibula and seven involved both bones (Table 3).

**Table 3:** Number and distribution of fractures by location and age group in a population-based cohort of 388 children (50.3% female) aged 0–2 years (birth related injuries not included)

Fracture location	0–6 months (*n* = 15)	6–12 months (*n* = 54)	12–18 months (*n* = 139)	18–24 months (*n* = 180)	Total (%)
Skull	3	4	1	0	8 (4.9)
Clavicle	0	10	8	8	26 (15.9)
Humerus	1	0	4	8	13 (8.0)
Ulna	0	0	3	2	5 (3.0)
Radius	0	1	9	13	23 (14.1)
Antebrachium	1	2	9	7	19 (11.6)
Metacarpal	0	0	1	0	1 (0.6)
Phalanx, hand	0	2	2	5	9 (5.5)
Ribs	1b	0	0	0	1 (0.6)
Spine	0	0	1a	0	1 (0.6)
Femur	1	4	1	4	10 (6.1)
Tibia	1	5	14	10	30 (18.4)
Fibula	0	0	3	1	4 (2.5)
Leg, both bones	0	3	3	1	7 (4.3)
Metatarsal	1	0	0	5	6 (3.7)
Total (%)	9 (5.5)	31 (19.0)	59 (36.2)	64 (39.3)	163 (100)

(a) Compression fracture to vertebra Th 11, after a fall from 3 m, (b) 11 rib-fractures

The number of fractures increased significantly by age group, with 9/163 (5.5%) fractures seen amongst 0-6-months-old, 31 (19.0%) in those aged 6–12 months, 59 (36.2%) in the 12-18-months-old and 64 (39.3%) in the 18-24-months-old (*p* = 0.027) (Table 3).

The distribution of fractures differed according to age group, with skull fractures predominating in 0-6-months-old (33.0%), clavicle fractures (33.0%) in 6-12-months-old and forearm fractures in the two oldest age groups (35.0% and 34.4%, respectively) (*p* < 0.001) (Table 3).

Fracture localization within the bone is provided in Table 4. 23/26 (88.5%) fractures to the clavicle involved the shaft. For the humerus, radius and tibia, most fractures were distal, whilst fractures to the femur most often involved the shaft.

**Table 4:** Location of fractures within the bone in 152 of the 163 fractures (fractures to the ribs and axial skeleton not included)

Main injury location	Proximal	Distal	Shaft	Total (%)
Clavicula	0	3	23	26 (17.1)
Humerus	3	7	3	13 (8.6)
Ulna	1	1	2	5 (3.3)
Radius	2	18	3	23 (15.1)
Antebrachium	1	12	6	19 (12.5)
Hand	0	2	0	2 (1.3)
Femur	2	3	5	10 (6.6)
Tibia	9	17	4	30 (19.7)
Fibula	0	1	3	4 (2.6)
Leg, both bones	2	5	0	7 (4.6)
Metacarpal	1	0	0	1 (0.7)
Phalanx hand	2	6	0	8 (5.3)
Metatarsal	3	2	0	5 (3.3)
Total (%)	26 (17.1)	77 (50.7)	49 (32.2)	152 (100)

### Types of fractures by age group

The fracture type varied according to age group (*p* = 0.126) (Table 5). Incomplete fracturs, e.g. greenstick/buckle/bowing fractures, were prevalent in all age groups, constituting 33.3% of fractures in 0-6-month-olds, 32.3% in 6-12-month-olds, 54.2% in 12-18-month-olds and 51.6% in 18-24-month-olds. Complete fractures were most often seen in infants aged 6–12 months, accounting for 16 cases (51.6%) as compared to 32.7% of those between 12 and 18 months (Table 5). Two cases were defined as inconclusive due to uncertain findings and/or low-quality imaging. These were still treated as fractures because of the strong clinical suspicion. No epiphyseal separations or classic metaphyseal lesions (CMLs) were seen.

**Table 5:** Number and types of fractures by age group in a population-based cohort

Fracture type	0–6 months	6–12 months	12–18 months	18–24 months	Total (%)
Complete	3	16	19	26	64 (39.3)
Incomplete /greenstick/buckle, bowing)	3	10	32	33	78 (47.9)
Avulsion	0	0	1	2	3 (1.8)
Fissure	3	5	6	2	16 (9.9)
Inconclusive/not defined	0	0	1	1	2 (1.2)
Total (%)	9 (5.5)	31 (19.0)	59 (36.2)	64 (39.3)	163 (100.0)

### Time between injury and medical attendance

The time interval between sustaining a fracture and seeking medical assistance at the A&E department ranged from 0 to 21 days, with a mean of 1.7 days (SD = 3.3). 39.9% of the patients with a fracture sought medical attention on the same day as the injury occurred, while 29.4% presented on day 2, 4.9% on day 3, and 4.3% on day 4. On the fifth day, 3.1% of cases were recorded, while 1.2% of cases turned up after six days. The seventh and eleventh day contributed for 1.8% each. Amount and frequency of cases decreased with longer time durations. The longest time interval of 21 days constituted for one single case (0.6%). In 9 cases (5.5%), the time interval was unknown, and in 7 cases (4.3%), no information was documented in the available medical records.

### Healing fracture

In 18/163 fractures (11.0%) there were radiological signs of healing at time of diagnosis. In four out of these cases, there was a mismatch between the reported time of injury and the appearances of the fracture, or no history of an injury; one 20-month-old with a fracture to the femur diaphysis; two one-month-olds of whom one had 11 rib fractures and multiple subdural hematomas and one had a fracture to the shaft of the tibia and one 16-month-old with a distal tibia fracture which was diagnosed at follow-up due to an avoidance response, but no history of a trauma.

In 3 of the 18 cases presenting with a healing fracture, two were subsequently diagnosed with non-accidental injury whilst one was further investigated for a generalized bone disease without conclusion.

### Children with a suspicion of Non-Accidental Injury (NAI)

To identify cases of suspected NAI, we noted children with fractures having had a skeletal survey during the same visit, according to protocol. A total of 19 children (10 females and 9 males) were identified, of which 16 had a skeletal survey because of a suspected inflicted injury, one due to a high-energy trauma (fall from 3 m) and two due to suspicion of an underlying bone disease. Based on the provided history, radiological findings and subsequent clinical evaluation, four children (one male, 3 months of age) and three females (3, 4 and 12 months of age, respectively) were subsequently believed to have sustained an inflicted injury, all of whom had undergone a skeletal survey during their first visit to the A&E. All four had radiological “red flags” for NAI, of whom two had all three features (fracture healing signs, unknown fracture mechanism, delay in seeking medical assistance).

### Associations between radiological “red flags” and a diagnosis of NAI

**Fig. 3:**
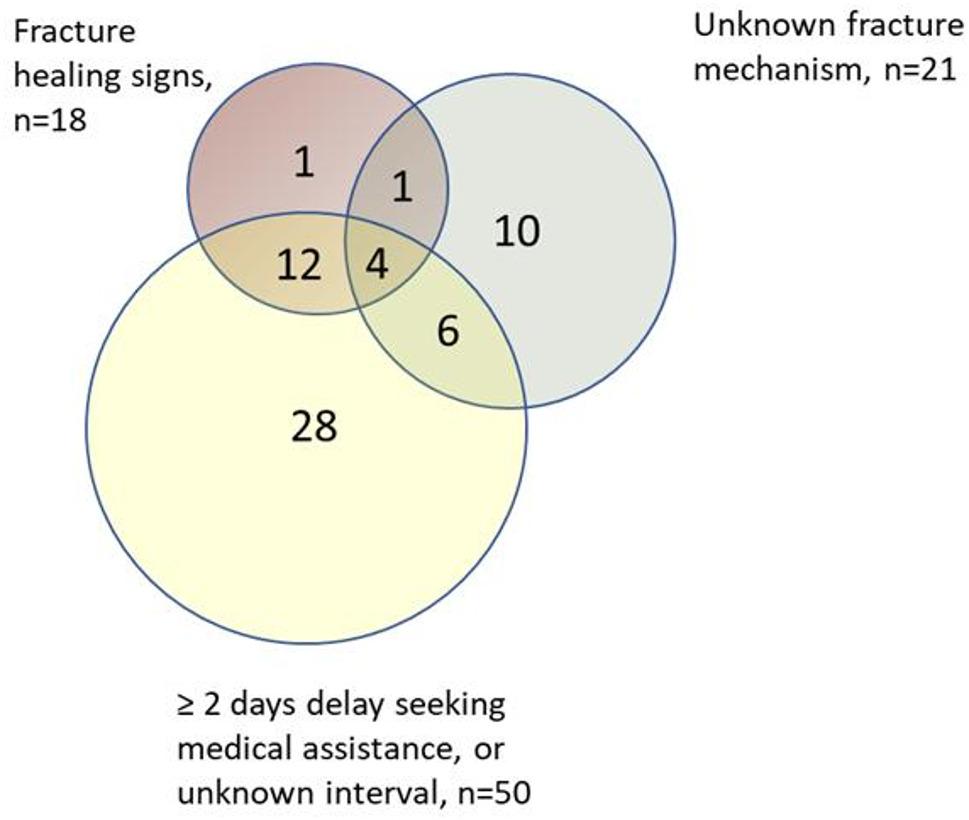
Triple Venn diagram identifying sheared features known to be associated with inflicted injury (fracture healing signs at time of diagnosis, unknown fracture mechanism and an unexpected delay in seeking medical assistance, set at ≥ 2days or an unknown interval)

Figure 3 shows a triple Venn diagram, identifying some sheared features known to be associated with inflicted injury, namely fracture healing signs at time of diagnosis (*n* = 18), an unknown fracture mechanism (*n* = 21) and an unexpected delay in seeking medical assistance, here set at ≥ 2 days after the sustained fracture or an unknown interval (*n* = 50).

Four children, 3 males aged 3 months, 17 months and 21 months, respectively, and one girl aged 3 months, shared all three features, of whom two (both 3 months old) turned out to have sustained inflicted injury and the other two had fractures to the fibula shaft (one incomplete and one complete). For the remainder of those with “red flags”, further investigation revealed NAI in two. It was beyond the scope of this work to elaborate more on this issue.

### Birth related injury

Forty-two children sustained a birth-related injury, of whom 21 (50.0%) had a fracture, yielding a fracture-incidence of 1.1 per 1,000 live born. Fractures to the clavicle (*n* = 10) and humerus (*n* = 10) were the two most common. In addition, there was one femur fracture that was diagnosed five days after a difficult labor (without any signs of healing). Most birth related fractures were examined due to clinical suspicion, before leaving the maternity unit.

## Discussion

We have shown, using a population-based design, that the incidence of fractures amongst 0-2-year-olds is relatively low, at 4.3 per 1,000 children, varying from 2.4 per 1,000 in infants to 6.1 per 1,000 in 1–2-year-olds, with no differences according to gender. Moreover, the distribution of fractures differs according to age, with skull fractures predominating in 0-6-month-olds, clavicle fractures in 6-12-month-olds and forearm fractures in 12-24-month-olds. Similarly, injury mechanism differs according to age, with falls from more than own height/furniture predominating in infants and fall from own height/crush injuries predominating in those 12–24 months of age. Of note is the low occurrence of injuries amongst 0-6-month-olds, and the fact that none of the 430 patients, birth injuries included, sustained a CML. The incidence of birth injuries was 1.1 per 1,000 live-born babies.

In a recent systematic review, including a total of 12 studies, the annual fracture incidence, birth injuries excluded, was reported at 5.3–9.5 per 1,000 children aged 0–2 years, which is slightly higher than our result of 4.3 per 1,000 [4]. Interestingly, children living in urban areas had a higher annual fracture incidence as compared to those living in rural areas, 5.2 vs. 2.5 per 1,000, which, to the best of our knowledge, is a finding not previously reported in young children. Several factors, including activity patterns, environmental hazards and healthcare accessibility may contribute to these differences, however, we cannot conclude based on our study. Several studies have addressed this issue in older children, with diverging results. Some studies have reported on higher fracture incidences amongst rural pediatric populations [8–11], rural patients having more severe injuries with most being motor vehicle accidents. The authors found that differences were associated with activity at injury and discussed the possibility that this effect was due to the influence of place activity patterns.

The distribution of fractures seen in our study, with clavicle fractures predominating in 6-12-month-olds and forearm fractures in 12-24-month-olds, compare well with the results of the recent systematic review, reporting that the most common sites were the radius/ulna (25–40%), followed by the tibia/fibula (17–28%) and the clavicle (15%) [4]. In comparison, we found that nearly 29% of the fractures were confined to the forearm and 25% involved the leg.

More importantly, and in line with previous literature, none of the 430 0-2-year-olds in our study sustained a CML, indicating that this type of lesion must be due to a special injury mechanism. According to the literature, CMLs are caused by traction/torque, and should raise the concern for non-accidental injury (NAI), instigating further investigations by the Child Protection Services [5]. However, iatrogenic causes have increasingly been reported, most often as isolated lesions in relation to a birth injury [12] or after manipulation of infant limbs in certain medical settings [13]. Interestingly, we found no CMLs in our study, which also included birth injuries and high energy trauma. One might speculate that CMLs might be occult, however, the published short reports/case reports describe clinical findings and/or child discomfort associated with this type of injury [14].

In terms of fracture mechanism, fall from more than own height/furniture constituted 23.9% of all the fractures and predominated in infants, whilst fall from own height constituted 17.3%, predominating in those 12–24 months of age. Again, this finding compares well with four of the studies included in the systematic review, with fall from chair, bed, table, own height or fall following indoor activity causing 50–60% of fractures [4].

In the present study, we evaluated the association between injury mechanism and occurrence of fracture. As one might expect, more than 70% of children falling from above their own height sustained a fracture, as compared to 42% of those falling from own height.

Moreover, children dropped by their parents had a high fracture rate of more than 60%, one third of fractures involving the femur. In total ten femur fractures, of which four were incomplete, were identified, five amongst infants and five amongst 12-24-month-olds. Contrasting a population-based study from 2022, reporting five femur fractures, all located distally, only one third of the femur fracturs in our series involved the distal part of the bone [6]. Further, and in line with others, around 70% were caused by fall from more than own height or dropped by parents [2, 15, 16]. According to the existing literature, a child sustaining a femur fracture has approximately a 1 in 3 chance of having been abused, and femur fractures resulting from abuse are more commonly seen in children who are not yet walking [5].

Interestingly, we found a relatively high occurrence of trampoline injuries, with a fracture rate of nearly 45%, mainly located in the tibia of children aged 18–24 months. Nearly half were in the proximal tibia, while two were located distally and two were involving the shaft. According to the literature, the incidence of trampoline fractures has increased significantly during the past 20 years and is often seen as a transverse fracture of the proximal tibia [17]. A large, population-based study including 101 trampoline-related fractures found a mean age of three years 10 months (SD 1.6 years) [17]. To the best of our knowledge, our study is the first to report population-based numbers on trampoline injures/fractures in 0–2-year-olds [17].

In this study, 19 children had an additional skeletal survey later the same day, of which 16 were performed due to a suspicion of inflicted injury. From a radiological point of view, signs of fracture healing, an unknown injury mechanism, and an unexplained delay in presenting to an A&E department are known “red flags” for NAI. A fourth “red flag”, namely inconsistency between radiological findings and the history provided by the caretaker, was difficult to assess precisely due to the retrospective study design.

By using a triple Venn diagram, we identified four children, three males and one female, having three features known to be associated with inflicted injury, of whom two 3-months-old subsequently were diagnosed with NAI. It is beyond the scope of this work to detail the rationale behind the NAI-diagnosis. The other two, aged 17 and 21 months respectively, had sustained fractures to the fibular shaft only. Both suffered minor discomfort, resulting in a 6–8-day delay in seeking medical attention; both injuries were unwitnessed, and both fractures showed signs of healing. All in all, the chain of events, with an unwitnessed trauma leading to mild discomfort, a delayed diagnosis and fracture healing signs, cannot be perceived as “red flags”, but as a natural course of a fracture to the fibula.

Of the 19 children having two “red flags”, one was diagnosed with NAI, whilst the fourth NAI-case, 12 months old, presented with a skull fracture and subdural hematomas after an alleged minor trauma. For the rest of those having “red flags” there were natural explanations. Interestingly, of the 16 skeletal surveys performed for suspected inflicted injury, a diagnosis of NAI was found in 25%.

In our study we found a relatively low incidence of birth-related fractures, 1.1 per 1,000 live births. Most of the fractures involved the clavicle or humerus, a finding in line with others [18].

We acknowledge some limitations to our study. First, its retrospective nature, with potentially incomplete or missing data. Second, we aimed at including only those residing in the municipalities of Tromsø. During the study period, a few were moving, and some of those residing in the southern part of the area might have attended a hospital outside the area under investigation. However, based on the population pattern within the municipalities studied, we do not believe that this has biased our results. The main strengths of the study are its population-based design, including both high energy trauma and birth-related injuries in children under two years of age, the meticulous collection and analysis of radiological examinations and the homogenous population.

## Conclusion

Injuries and fractures in children under two years of age are rare, particularly in infants. The occurrence of radiological “red flags” should raise suspicion of non-accidental injury. No CMLs were identified in this population-based cohort, indicating that this type of fracture has a specific fracture mechanism. Trampoline injuries are associated with a high fracture rate in children aged 18–24 months.

## Data Availability

Data are available on specific request.

## Abbreviations

A&E department: Accident and Emergency department
SD: Standard deviation
CML: Classic metaphyseal lesion
SNBF: Subperiosteal new bone formation
ANOVA: Analysis of Variance
NAI: Non-accidental injury
